# Photochemical control of bacterial gene expression based on *trans* encoded genetic switches†

**DOI:** 10.1039/d0sc05479h

**Published:** 2021-01-12

**Authors:** Avishek Paul, Jingyi Huang, Yanxiao Han, Xintong Yang, Lela Vuković, Petr Král, Lifei Zheng, Andreas Herrmann

**Affiliations:** Zernike Institute for Advanced Materials, Dept. of Polymer Chemistry, University of Groningen Nijenborgh 4 9747 AG Groningen The Netherlands zhenglifei0926@gmail.com; DWI-Leibniz Institute for Interactive Materials Forckenbeckstr. 50 52056 Aachen Germany herrmann@dwi.rwth-aachen.de; Department of Chemistry, University of Illinois at Chicago Chicago Illinois 60607 USA; Department of Chemistry, University of Texas at El Paso El Paso Texas 79968-0513 USA; Department of Physics, University of Illinois at Chicago Chicago Illinois 60607 USA; Department of Biopharmaceutical Sciences, University of Illinois at Chicago Chicago Illinois 60612 USA; Institute of Technical and Macromolecular Chemistry, RWTH Aachen University Worringerweg 2 52074 Aachen Germany

## Abstract

Controlling gene expression by light with fine spatiotemporal resolution not only allows understanding and manipulating fundamental biological processes but also fuels the development of novel therapeutic strategies. In complement to exploiting optogenetic tools, photochemical strategies mostly rely on the incorporation of photo-responsive small molecules into the corresponding biomacromolecular scaffolds. Therefore, generally large synthetic effort is required and the switching of gene expression in both directions within a single system remains a challenge. Here, we report a *trans* encoded ribo-switch, which consists of an engineered tRNA mimicking structure (TMS), under control of small photo-switchable signalling molecules. The signalling molecules consist of two amino glycoside molecules that are connected *via* an azobenzene unit. The light responsiveness of our system originates from the photo-switchable noncovalent interactions between the signalling molecule and the TMS switch, leading to the demonstration of photochemically controlled expression of two different genes. We believe that this modular design will provide a powerful platform for controlling the expression of other functional proteins with high spatiotemporal resolution employing light as a stimulus.

## Abstract 2

Controlling gene expression by light with fine spatiotemporal resolution not only allows understanding and manipulating fundamental biological processes but also fuels the development of novel therapeutic strategies.

## Graphical abstract

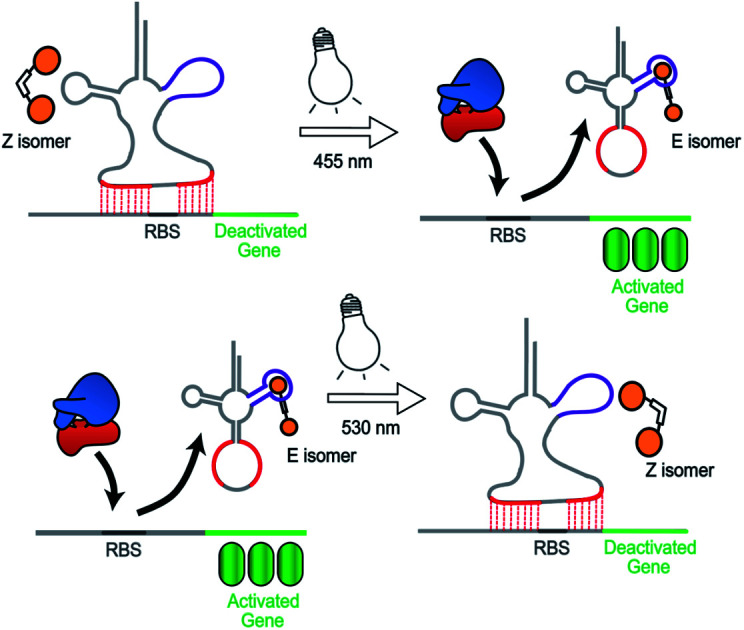

## Introduction

Synthetic biology aims to develop genetic circuits to reprogram cell behaviour.^1,2^ These complex genetic parts that allow perturbing and interpreting biological processes usually function through reacting to exogenous chemical inducers.^3,4^ However, these chemical inducers generally result in a constitutive effect, *i.e.*, in a continuous “ON” state, which limits their application to study inherently dynamic behaviour of cells.^5^ In contrast, light can be considered as an excellent tool that matches those dynamics due to its high spatiotemporal resolution, complete bio-orthogonality and fine tunability in regard to its wavelength and intensity.^6,7^ To date, two approaches have been widely used to control gene expression using light. One is the application of optogenetic tools, which generally control protein expression by regulating the interaction between a photo controllable transcription factor and its promoter through light irradiation.^8,9^ This approach necessitates the expression of additional photo-responsive proteins which might cause burden on metabolic and cell signaling pathways.^10^ The second photochemical approach^11^ relies on the installation of light responsive small molecules onto the bio-macromolecular scaffolds including nucleic acids^12,13^ and proteins,^14–16^ thereby providing an extra layer of control over their biological functions by light. This approach generally requires large synthetic efforts for covalently modifying the bio-macromolecules with photo-caging groups^17^ or photo-switches.^18,19^ Moreover, the utilization of photocages only permits a single-way gene regulation event due to the deprotective removal of the caging groups under light illumination.^20^

**Fig. 1:**
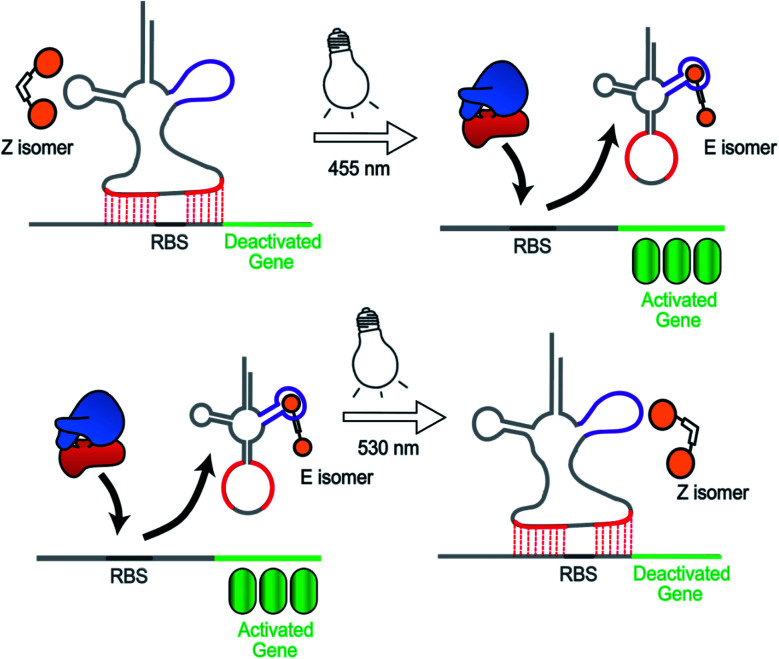
Concept to control gene expression by light employing the TMS switch and photo switchable ligands. The two photo-isomers exhibit different affinities to the sensory element of the TMS switch and hence allow photo-control of gene expression. The two isomers can be converted into each other by light and this structural change is enabled by an azobenzene moiety indicated by a white box connecting two ligands (shown in orange) that bind to the TMS switch in the magenta region.

To overcome these limitations, we herein present a novel photochemical approach to control bacterial gene expression based on a recently developed *trans*-encoded genetic switch,^21^ which binds both flanking sites of the ribosome binding site (RBS) of the target mRNA, without disturbing the RBS and the gene of interest, to block ribosome entry and subsequent translation. Moreover, since RBS flanking sites do not contain any specific nucleotides, the same switch can target any gene without any alteration of the gene sequence. To reverse repression, we firstly employed an anti-repressor RNA that binds the tRNA mimicking structure (TMS) and as a consequence pulls the TMS off from the mRNA, liberating the RBS to induce gene expression. Moreover, the modular TMS was reengineered to be responsive to other inputs than RNA. Bacterial tRNA structure harbors a loop containing the dihydrouridine base. This loop is called D-loop. With the replacement of the D-loop of TMS switch by a Neomycin B aptamer sequence,^22,23^ a RNA switch was obtained, which responds in a concentration-dependent manner to a Neomycin B derivative as input. Moreover, it was found that the ligand mediated gene switching was highly depending on the affinity of the ligand toward the aptamer component of the switch. Inspired by this observation, we aimed to engineer the gene switch to be photo-responsive by fabricating a ligand bearing a photo-switchable unit that can undergo a light-induced structural change to exhibit two photo-isomers. We reasoned that these isomers would have different binding affinities and/or binding modes towards the sensor domain of the TMS switch and thus can induce photo-control of protein expression (Fig. 1). In contrast to the photocaged systems, our design aims to control gene expression by regulating the noncovalent interactions between ligand and an aptamer domain on the riboswitch by light, and thus have the added benefits of being synthetically easily accessible and capable of regulating translation in both directions – from OFF to ON and from ON to OFF.

## Results and discussion

### Design of the photo-switchable ligand binding to the TMS switch

**Fig. 2:**
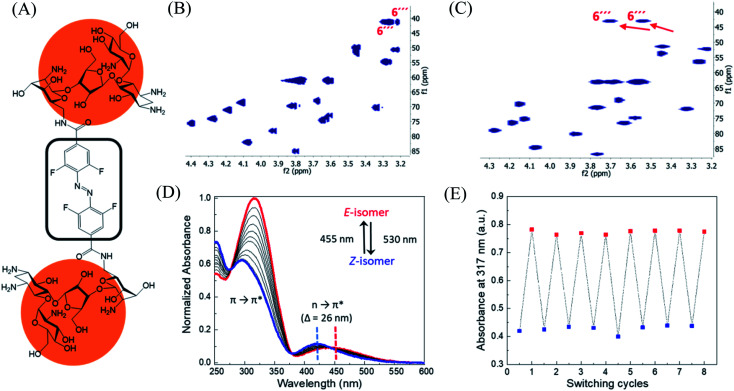
Structure and characterization of F-dimers by linking two paromomycin units with a difunctional azobenzene core. (A) Chemical structure of F-dimer. (B) HSQC spectrum (500 MHz, D_2_O) of paromomycin. (C) HSQC spectrum (500 MHz, D_2_O) of F-dimer (see Fig. S3† for full spectrum). Red arrows indicate the shift of specific signals caused by the regioselective transformation of the amino group in position 6′′′ of ring IV: *J*(C6′′′–H_a_) and *J*(C6′′′–H_b_) coupling. (D) Changes in the absorption spectra of a solution of F-dimer in MQ water (10 μM) upon irradiation at 530 nm (*E* → *Z*) and at 455 nm (*Z* → *E*). Blue and red dashed lines indicate the separation of n → π* bands between the two isomers of the F-dimer. (E) Repetitive switching cycles of F-dimer upon alternating irradiation with green light (*λ* = 530 nm) and blue light (*λ* = 455 nm). After eight cycles, no fatigue indicated by a reduction of absorbance was observed. The absorbance was measured at the maximum of the π–π* transition (317 nm) of the *E*-isomer.

In order to design the photo switchable ligand binding to the TMS switch, we decided to take paromomycin as the parent compound for further modifications. The reasons for this choice are twofold. First, paromomycin exhibits high binding affinity against the Neomycin B aptamer (*K*_d_ = 0.2 μM, Fig. S1†) as determined by isothermal titration calorimetry (ITC).^24^ This binding is even stronger than that between the same sequence and NeomycinB-azide (*K*_d_ = 28 μM), which was used as input to control gene expression in a previous study.^21^ Second, given that paromomycin has a unique aminomethyl group with little steric hindrance, it can be readily functionalized by activated acylating reagents with high regioselectivities.^25^ With these two considerations in mind, a type of photo-switchable dimeric paromomycin (Fig. 2A) containing *o*-fluoroazobenzene moiety (F-dimer) as the photo-responsive linker was designed. The introduction of fluorine atoms in the *ortho*-positions not only allows the use of light in the visible range (*λ* > 500 nm) instead of UV light to trigger the *E* → *Z* isomerization, but also dramatically enhances the stability of the thermodynamically unfavorable *Z*-isomers.^26,27^ We hypothesized that the *Z*-isomers would bind less efficiently to the TMS switch since the amino sugar rings in the *Z*-isomers are arranged in a more crowded configuration than in the *E* isomer, thus introducing steric hindrance and eventually blocking the binding to the aptamer domain of the TMS switch.

### Synthesis and characterization of F-dimer

Following our design, the photo-switchable F-dimer was synthesized from 2,2′,6,6′-tetrafluoroazobenzene-4,4′-dicarboxylic acid by well-established amide bond formation procedures^27^ (Scheme S2†). This compound was activated to form the corresponding *N*-hydroxysuccinimide (NHS) ester, which was reacted with two aminoglycoside units. In this way, the aminomethyl group in position 6′′′ of paromomycin was functionalized by activated acylating reagent with high regioselectivities^25^ forming the photo-switchable F-dimer. The crude product was purified by high performance liquid chromatography (HPLC) and characterized by 1D- and 2D-NMR (heteronuclear single quantum coherence (HSQC)) spectroscopy (Fig. S2 and S3†). As shown in Fig. 2C, the HSQC spectrum of the F-dimer shows a remarkable shift of the *J*(C6′′′–H) coupling of ring IV in comparison to the 2D-spectrum of pristine paromomycin (Fig. 2B) proving the regioselective acylation reaction in the C6 position of ring IV.

The photo-physical properties of F-dimer in water were studied using UV-vis spectroscopy. The UV-vis spectrum of the *E*-isomer of the F-dimer exhibits a characteristic strong π → π* absorption band at short wavelength (*λ* = 317 nm) and a weaker n → π* band centered around 446 nm (Fig. 2D). Fluorine substituents in the *ortho*-positions, as σ-electron withdrawing groups, can lower the n-orbital energy of the *Z*-isomer, resulting in a blue-shift of the n → π* absorption band for the *Z*-isomer.^26^ As a result, the two isomers of F-dimer exhibit a separation of n → π* bands of 26 nm, which allows to selectively trigger the *E* → *Z* isomerization with visible light at wavelengths longer than 500 nm. Thus, the solution was irradiated with a 530 nm LED coupled to an optical fiber. During the *E* → *Z* photo-isomerization hypochromism and bathochromic shifts were concomitantly observed in the region of π → π* absorption, alongside with an increase in absorbance coupled with shift towards shorter wavelengths in the region of n → π* absorption. During the *Z* → *E* photo-isomerization upon irradiation at 455 nm, the π → π* absorption peak shifted from 295 nm to 317 nm while the n → π* absorption peak shifted from 420 nm to 446 nm until reaching the thermodynamic equilibrium state, also known as photo-stationary state. Both photo-isomerization processes exhibit three isosbestic points at 276 nm, 378 nm and 442 nm.

Using 1D-NMR the ratio of the two photo-isomers of F-dimer at the photo-stationary state (PSS) were determined. Irradiation with visible light was used to isomerize F-dimer in both directions, producing PSSs containing 78% of *Z*-isomer with green light (530 nm) and 75% of *E*-isomer with blue light (455 nm) (Fig. S2a and b†). Multiple *E*/*Z* photo-isomerization cycles did not result in any noticeable degradation, highlighting the robustness of the F-dimer switch (Fig. 2E). As a consequence of the stabilization of the n-orbital of the *Z*-isomer through the introduction of *ortho*-fluoro substituents, thermal *Z* → *E* isomerization of F-dimer shows a half-life of more than 8 days at 37 °C in the dark (Fig. S6†). These photo-physical characteristics, *i.e.* irradiation at longer wavelength and high thermal stability, will be beneficial for controlling gene expression using light in living systems as shown below.

### Studies on the interactions between photo-isomers and the aptamer domain of TMS switch

**Fig. 3:**
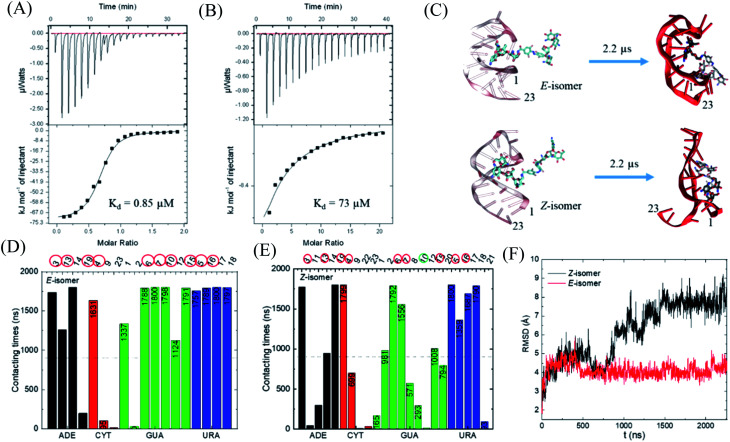
Study of the binding between the F-dimer isomers and the aptamer domain of the TMS switch. ITC titration curves of (A) *E* isomer (70 μM in 20 mM phosphate buffer pH 7.5) into aptamer solution (7 μM in 20 mM phosphate buffer pH 7.5) and (B) *Z* isomer (700 μM in 20 mM phosphate buffer pH 7.5) into aptamer solution (7 μM in 20 mM phosphate buffer pH 7.5); (C) initial and final snapshots for the *E*/*Z*-isomers with RNA aptamer (the nucleotides are numbered from 5′ to 3′ as 1 to 23); (D) contacting times of nucleotides with *E*-isomer during simulation. (E) Contacting times of nucleotides with *Z*-isomer during simulation. ADE = adenine, CYT = cytosine, GUA = guanine, URA = uracil; (F) RMSD for the nucleotides forming the initial binding pocket in the presence of *E*- or *Z*-isomer.

With this photo-switch at hand, we firstly determined the binding affinity of the *E* and *Z* F-dimers against the sensor domain of the TMS switch. For this purpose, the isomers of the resulting F-dimer were isolated by HPLC with >95% purities (Fig. S2c and d†) thanks to their extreme thermal stability. The purified isomers and a 23 mer aptamer^22,23^ that will be later incorporated into the TMS switch structure were then used for isothermal titration calorimetry (ITC) studies. It was observed that the two isomers can bind to the sensor domain with different affinities – the *E*-isomer shows significantly higher binding affinity (*K*_d_ = 0.85 μM, Fig. 3A) than that of the *Z*-isomer (*K*_d_ = 73 μM, Fig. 3B) against the sensor domain. This result supports our hypothesis that the more crowded configuration of *Z*-isomer prevents its effective binding to the aptamer.

To obtain a better insight into the nature of interactions between the photo-isomers and the aptamer, we carried out atomistic molecular dynamics (MD) simulations of the binding between *E*/*Z*-F-dimer and the 23 mer aptamer sequence by NAMD^28^ and the CHARMM force fields.^29,30^ The structure of the aptamer was taken from the PDB that was previously determined by NMR.^31^ The particle mesh Ewald (PME)^32^ method was used for the evaluation of long-range coulombic interactions. Fig. 3C shows the initial and final states of each simulation (for the trajectories of F-dimers interacting with aptamer, see Fig. S7†). Initially, only one paromomycin of the F-dimers was inserted in the binding pocket on the aptamer (Fig. 3C left column). The *Z*-isomer causes severe conformational changes of the aptamer, while the *E*-isomer induces minor disturbance in the aptamer structure (Fig. 3C right column). In the case of the *Z*-isomer, the first paromomycin, which initially binds to the aptamer groove, gets distorted and shifts away from the initial binding pose, while the second paromomycin opens up the RNA groove due to competitive binding to the RNA backbone caused by the bent structure preserved by the *Z*-azobenzene. In contrast, for the *E*-isomer, the first paromomycin stays more anchored in the RNA groove, while the second paromomycin transiently binds to the RNA or stays solvated in the solution due to the *trans*-configuration, which perturbs the aptamer structure less.

Next, we analyzed the contacting nucleotides with *E*/*Z*-F-dimer. The nucleotide is considered to interact with F-dimers if it is within 3 Å of the *E*/*Z*-isomer. The number of contacting nucleotides in the *Z* case plummets at 1.75 μs, when the RNA groove opens significantly, while the number for *E*-isomer remains similar during the whole simulation time (Fig. S8†). We further analyzed the contact times of each nucleotide over the last 1.8 μs. The nucleotides which interact less than half of the total time are considered to be less involved in binding. The initial nucleotides forming the binding pocket were encircled red in Fig. 3D and E. Nucleotides out of the initial binding pocket also show stable binding to the *E*/*Z*-isomers due to re-adjustment of binding during simulation. In the *Z* case, one of the initial nucleotides (#10) marked in green shows reduced binding times, while all the initial nucleotides in the *E* case participate in the binding strongly. The total number of interacting nucleotides in the *E* case (18) is less than that of the *Z*-isomer (23). Besides, the contacting time shows bipolarity among the nucleotides in the *E* case, meaning that the nucleotides are binding either for long or very short times during the whole simulation process, while it is in a more random order in the *Z* case (Fig. 3D and E).

The binding energy between F-dimers and aptamer was calculated by VMD plugin^33^ (Fig. S9†). The total binding energy is more waved in the *Z*- than in the *E* configuration. Moreover, the total binding energy in the *Z* case fluctuates up at 1.75 μs due to opening up of the RNA aptamer. The average binding energy (total) for the *Z* isomer after 1.75 μs is −1602 kcal mol^−1^, while it is −1701 kcal mol^−1^ for the *E* isomer, which indicates a stronger binding between the *E* isomer and the aptamer. Fig. 3F shows the root-mean-square deviation (RMSD) for the initial nucleotides encircled in Fig. 3D and E. The *Z*-isomer breaks the initial pocket indicated by the big fluctuation of the RMSD value; in contrast, the *E*-isomer only slightly disturbes the structure of the initial pocket.

In summary, during binding of the *E* isomer the “canonical” strong neomycin B – aptamer binding mode is maintained, whereas the *Z* isomer does not resemble the initial binding mode. The different binding modes of the two different isomers originate from the structural changes induced by *E*/*Z*-azobenzene moieties. The *Z*-isomer deforms the groove of the RNA aptamer and binds in a non-specific and weak manner, while upon binding of the *E*-isomer the specific binding mode is preserved due to the larger distance between the two paromomycin units. Based on these results, we anticipate that the *Z*-isomer would bind less than the *E*-isomer to the aptamer domain of the TMS switch and thus is unable to turn on protein production. Accordingly, control over gene expression by light can be achieved by switching the conformation of the photo-isomers and their interactions with the TMS switch.

### Photochemical control over gene expression with F-dimer

After verifying the difference in binding between the photo-isomers against the sensor domain of the TMS switch, we seek to exploit the F-dimers to photochemically control gene expression in bacteria. Since the parent compound paromomycin is an antibiotic drug, we first examined the toxicity of the F-dimers in bacteria through a MIC test.^34^ It was found that both photo-switchable aminoglycoside isomers show no difference in antibacterial activity. Both exhibit a similar MIC value of >64 μM (Fig. S10†). Noteworthy, modifying paromomycin with a photo-switchable unit reduces its original antibacterial activity, which is a prerequisite for using it as a non-toxic input signal to control gene expression.

**Fig. 4:**
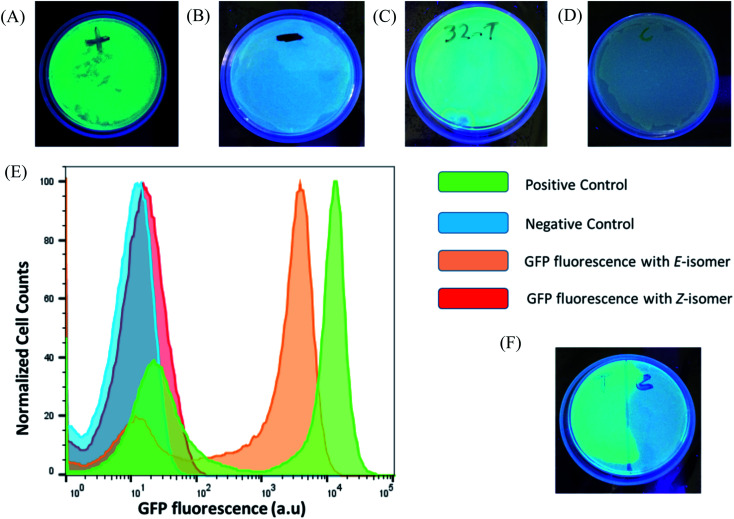
Photochemical control over GFP expression with F-dimer isomers. (A) Fluorescent images of the plates after overnight incubation at 37 °C: positive control (containing inducer to express GFP only without having any F-dimer); (B) negative control (containing neither inducers nor any F-dimer); (C) in the presence of 32 μM purified *E*-isomer; (D) in the presence of 32 μM purified *Z*-isomer. (E) Flow cytometry histograms of the GFP expression occurring on the plates from (A) to (D). The histograms show bimodal populations of GFP expression for the positive control and cells containing *E*-isomer. (F) Fluorescent image recording of *in situ* activation of GFP expression where a photomask was placed on the right half of the plate.

To prove the feasibility of our concept, HPLC purified *E*- and *Z*-F-dimers were employed to regulate GFP expression in bacteria. The TMS switch and the GFP gene were expressed from two separate plasmids in *Escherichia coli* BL21(DE3) cells. The TMS switch was produced from an IPTG inducible T7 promoter and the GFP mRNA was transcribed from an arabinose inducible promoter. The cells were transformed with the plasmids encoding the switch and GFP and were grown in LB medium in presence of ampicillin and chloramphenicol. In the meantime, LB agar plates were prepared supplemented with these two antibiotics corresponding to the plasmids used in this study and inducers to express the switch and the GFP mRNA. When the transformed cells in the LB medium reached the early log phase (O.D. = 0.4), inducers to express the switch and the GFP were added into the medium. Later, when the O.D. of the cells reached 0.8 they were spread on two LB agar plates. Then 32 μM purified *E*- and *Z*-isomer of the F-dimer were added onto these plates, respectively. Simultaneously, two other plates were prepared as a positive control (plate containing inducer to express GFP only without adding any F-dimer, Fig. 4A) and the negative control (plate containing neither inducers nor any F-dimer, Fig. 4B). All the plates were kept in the dark at 37 °C overnight. The results demonstrated that the *E*-isomer could successfully initiate GFP expression to the level similar to the positive control (Fig. 4C), while *Z*-isomer only induced very weak gene expression (Fig. 4D). This observation bolsters our previous hypothesis – *E*-isomer could effectively bind to the sensor domain of the TMS switch, distort the switch structure resulting in detachment of the TMS switch from the target GFP mRNA and consequently initiation of GFP translation. Due to the crowded configuration of the two paramomycin units and the lower affinity, *Z*-isomer was unable to trigger the TMS switch as input. To quantify the GFP expression on each plate, we dispersed the bacterial colonies in PBS buffer for flow cytometry analysis. These experiments showed that the positive control as well as the sample containing the *E*-isomer showed two populations of cells, one with a very low level of GFP expression and another with a high GFP expression level (Fig. 4E). We calculated the GFP_*E*_/GFP_*Z*_ ratio, which turned out to be as high as 110 fold. GFP_*E*_ signifies the GFP expression of the cells occurring in presence of the switch and the *E*-isomer while GFP_*Z*_ refers to the GFP expression in presence of the switch and the *Z*-isomer.

Next, to determine the effect of the concentration of the *E*-isomer on the GFP expression pattern, we incubated the cells with the pure *E*-isomer employing different concentrations ranging from 64 μM to 4 μM. These experiments indicated that the *E*-isomer can initiate GFP expression at 32 μM but not 16 μM (Fig. S11†). To more precisely determine the turn on concentration of GFP expression three more concentrations of the *E*-isomer in between 16 and 32 μM were investigated, *i.e.* 20, 24 and 28 μM. GFP expression was not visible at 20 μM, but when 24 μM and 28 μM of *E*-isomer were applied (Fig. S12†). This observation indicates that the TMS switch is highly sensitive to the concentration of the small molecule inputs.

Encouraged by these results, we intended to carry out the photo-isomerization of F-dimer *in situ* to control the bacterial gene expression. First, *Z* to *E* isomerization was studied. Following the procedures described above, 32 μM pure *Z*-isomer was added onto six plates with bacteria. Five of the plates were irradiated with light of a wavelength of 455 nm for 5 min, 10 min, 30 min, 1 h and 1.5 h, respectively, while the last plate was kept in the dark. Afterwards the plates were incubated at 37 °C overnight. As shown in Fig. S13,† the minimum exposure time required to partially turn on gene expression was 30 minutes and the plate that was treated with light for 1.5 hours showed maximum GFP expression. The ON/OFF ratio of this *in situ* switching was quantified to be around 112-fold. The ON/OFF ratio was calculated from the fluorescence of the GFP positive cells treated with light for 1.5 hours and fluorescence of the cells that were kept in dark. In contrast, the plate that was not illuminated with light and the plates that were irradiated for short periods of time showed no GFP expression. To confirm that the GFP expression was triggered only due to the *Z* to *E* isomerization but not because of any other effect of light irradiation, the same plate without *Z*-F-dimer was irradiated with light of a wavelength of 455 nm for 1.5 hours. It turned out that this plate did not show any GFP expression (Fig. S14†). These observations proved that the *Z*-isomer was converted to the *E*-isomer and subsequently triggered GFP expression. Moreover, the non-irradiated plate containing the *Z*-isomer did not show any GFP fluorescence as the *Z*-isomer itself is unable to trigger GFP expression through structural alteration of the TMS switch and liberation of the RBS on the mRNA.

Next, *E* to *Z* isomerization induced *in situ* to switch-off GFP expression was investigated. Two agar plates with transformed cells were prepared. Initially 32 μM pure *E*-isomers were added to these plates. One plate was irradiated with light of a wavelength of 530 nm for 1.5 hours and then both plates were kept at 37 °C for overnight incubation. It turned out that both plates showed similar level of GFP expression, suggesting that *E* to *Z* photo-isomerization was not as efficient as *Z* to *E* isomerization under the experimental conditions. We reasoned that the observed difference might originate from the presence of aptamer inside cells. To prove this, we performed an *in vitro* study where the photoisomerization of F-dimers was measured in the presence of Neomycin B aptamer. In this study, one equivalent of the photo-isomers was incubated with two equivalents of aptamer for one hour. Then, their photoisomerization processes were analyzed using UV-vis spectroscopy (Fig. S15†). It was observed that upon irradiation at 530 nm, the *E* → *Z* photoisomerization was significantly slowed down by the presence of RNA aptamer (Fig. S15A and B†). In contrast, under irradiation at 455 nm (*Z* → *E*), the presence of RNA aptamer barely influences the photoisomerization kinetics of F-dimers (Fig. S15C and D†). These results can be explained by the spatial confinement effect which plays a pivotal role in photoisomerization of azobenzenes.^35^ Our photo-switchable compounds are composed of two bulky paromomycin molecules linked by an azobenzene group and the compounds require a high degree of conformational freedom to switch between the two photo-isomeric forms. However, when the *E* isomer binds to the aptamer domain of the switch, the compound encounters a sterically hindered environment that confines space and hinders free rotation to convert the *E* to *Z* isomer.

Since the TMS switch is highly sensitive to the concentration of the *E*-isomer, we reasoned that to switch-off gene expression *in situ* might be achievable at a concentration that is close to the observed ‘threshold’ concentration where obvious GFP production started. To test this, the applied concentration of the *E*-isomer was reduced to 22 μM for the same experiments as described above. As indicated from the flow cytometry results (Fig. S16†), there was partial GFP fluorescence observed with 22 μM *E*-isomer but no GFP fluorescence was noticed when the plate was treated with 530 nm wavelength light, suggesting that a slight amount of transformation to the *Z*-form reduced the *E*-isomer concentration to cross the ‘threshold’ and thus effectively down-regulate the expression of GFP. Further improvement on the sensitivity of our system will be subject of future studies by either more elegant molecular design^36^ or increasing the intensity of the applied light.

As light as a stimulus holds great promise for locally activating a certain biological function, we next studied if the amalgamation of photo-responsive F-dimers and the TMS switch could spatially resolved address a certain population of bacteria on an agar plate. For this purpose, the transformed cells were spread on an agar plate and the *Z*-isomer was added on the plate. Then a photomask was placed to cover half of the plate. After 1 hour light irradiation (455 nm), the photomask was removed and the plate was incubated at 37 °C overnight. Fig. 4F shows that GFP was only expressed on the exposed part of the plate, while no GFP expression was observed on the other half of the plate where the photomask was originally placed. This experiment proves the capability of the F-dimer in combination with the TMS switch to realize spatial control over gene expression.

### Photochemical control over ϕX174 E lysis gene expression with F-dimers

**Fig. 5:**
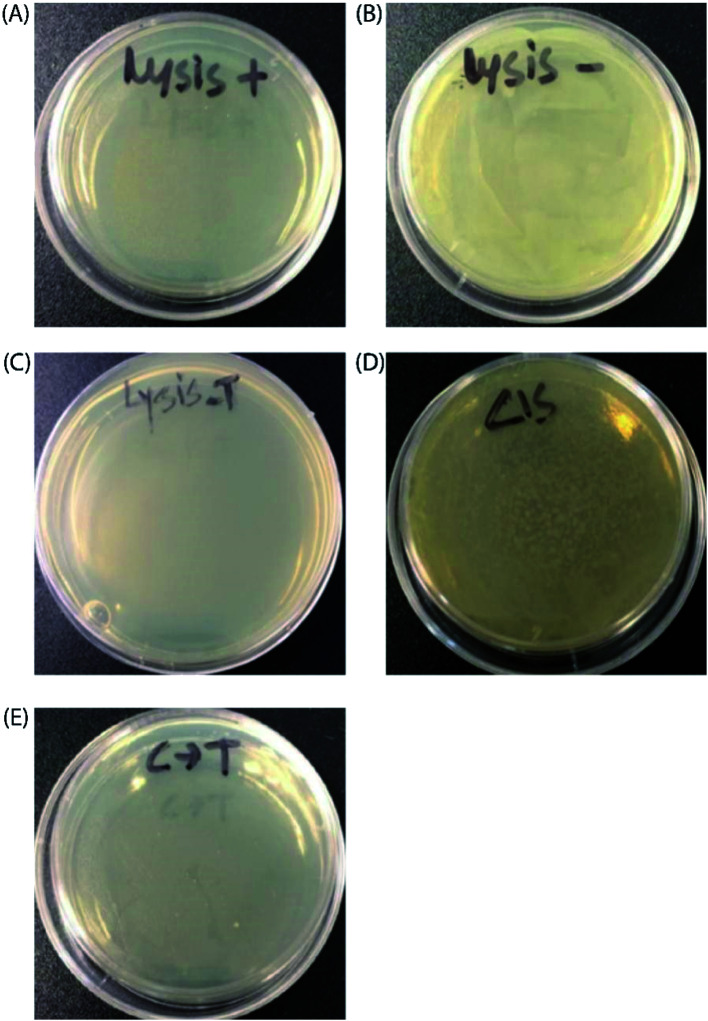
Photochemical control over ϕX174 E lysis gene expression with F-dimers. Images of the plates after overnight incubation at 37 °C: (A) positive control (containing inducer to express ϕX174 E lysis gene only without having any F-dimer); (B) negative control (containing neither inducers nor any F-dimer); (C) in the presence of 32 μM purified *E* isomer; (D) in the presence of 32 μM purified *Z* isomer. (E) *In situ* activation of ϕX174 E lysis gene expression by *Z* (*cis*) to *E* (*trans*) photoisomerization.

Finally, to prove the general applicability of our approach, we replaced the target GFP gene with a bacteriophage lysis gene (ϕX174 E) to photo-chemically control bacterial cell growth in the presence of the TMS switch and photo-switchable F-dimer. ϕX174 E lysis gene refers to a membrane protein of 91 amino acids, which hinders peptidoglycan synthesis in the bacterial cell wall and thus causes lysis of the host cell during growth.^37^ Following the same experimental design for controlling GFP expression, the TMS switch and the lysis gene were expressed from two separate plasmids in *Escherichia coli* BL21(DE3) cells. The cultured cells in which the expression of the bacteriophage lysis gene was initially inhibited by the TMS switch were spread on two agar plates. To these plates, 32 μM *E*- and *Z*-isomers were added, respectively. At the same time, two other plates were prepared as positive control (plate containing inducer to produce the lysis gene only, Fig. 5A) and negative control (plate containing both inducers but without any form of F-dimer, Fig. 5B). After overnight incubation at 37 °C, the plate treated with *E*-isomer did not show any bacterial growth, similar as the positive control plate (Fig. 5C). In stark contrast, the plate treated with *Z*-isomer was fully covered with bacterial colonies (Fig. 5D). Since we already demonstrated that the isomers of F-dimer showed no difference in antibacterial activity from the MIC test, the drastic difference of bacterial growth must be a result of the different level of lysis gene production induced by the two isomers. To further prove the photo control of this system, 32 μM *Z*-isomer was added to the agar plate with spread cells. Subsequently, the plate was illuminated with light of a wavelength of 455 nm for 1 hour and was kept overnight at 37 °C. The plate did not show any sign of bacterial growth (Fig. 5E). To rule out the effect of light, one plate was prepared containing transformed cells and *Z*-isomer but without addition of the inducer to express the lysis gene. Another plate with the same cells contained both inducers to express the TMS switch and the lysis gene but no *Z*-isomer. These plates were then illuminated with light of a wavelength of 450 nm for 1.5 h. After overnight incubation, both plates did not show any inhibition of bacterial growth (Fig. S17†), indicating the *in situ* switch-on of lysis gene expression originating from light induced conformational changes of the F-dimer.

## Conclusions

In summary, we have demonstrated a powerful photo-responsive gene expression system that relies on a TMS switch and the corresponding photo-switchable F-dimer ligand. The use of fluorinated azobenzene as a photo-responsive unit enabled our system to operate with visible light, which represents an important feature for future development in living systems. By switching the conformations of F-dimer using light, we were able to regulate their interactions with the aptamer domain of the TMS switch and accordingly up- or down-regulate the level of the target protein production. Importantly, this working mechanism was further validated with the help of ITC measurements and computational studies. Spatially resolved activation of gene expression was realized by applying light at the desired position. In addition, since our tRNA based TMS switch is *trans* encoded, it can easily be accommodated to control any other gene with the same photo-switchable ligand and the same TMS switch. This modularity and the general applicability of our system was demonstrated by expanding the control of GFP production to bacteriophage lysis gene expression. In this regard, it is worth to note that recent synthetic biology approaches are involving engineered bacteria to deliver drugs in eukaryotic cells.^38^ This opens the avenue of designing future photo-responsive gene expression systems where the on-site production of functional and therapeutic proteins can be controlled with high spatiotemporal resolution by light. Moreover, the TMS switch can be developed for photo-chemical systems in eukaryotic cells by replacing the bacterial tRNA scaffold with an eukaryotic tRNA structure. We believe that this implementation will raise broad interest in the fields of chemical and synthetic biology.

## Conflicts of interest

There are no conflicts to declare.

## Supplementary Material

SC-012-D0SC05479H-s001
