# Supplementary material for: Photochemical control of bacterial gene expression based on trans encoded genetic switches
Source: Chem Sci. 2021 Jan 12;12(7):2646–54. doi: 10.1039/d0sc05479h (PMC8179269; doi:10.1039/d0sc05479h)
Supplement: SC-012-D0SC05479H-s001 [file SC-012-D0SC05479H-s001.pdf]

Electronic Supplementary Material (ESI) for Chemical Science.
This journal is © The Royal Society of Chemistry 2021

Supplementary Information

Photochemical control of bacterial gene expression based on *trans* encoded genetic switches

Avishek Paul[1,2#,] Jingyi Huang[1#], Yanxiao Han[3#], Xintong Yang [1,2], Lela Vuković [4], Petr Král [3,5,6], Lifei Zheng [1,2,7]*, Andreas Herrmann [1,2,7]*

1 Zernike Institute for Advanced Materials, Dept. of Polymer Chemistry, University of Groningen, Nijenborgh 4, 9747 AG Groningen, The Netherlands.

2 DWI-Leibniz Institute for Interactive Materials, Forckenbeckstr. 50, 52056 Aachen, Germany.

3 Department of Chemistry, University of Illinois at Chicago, Chicago, Illinois 60607, USA.

4 Department of Chemistry, University of Texas at El Paso, El Paso, Texas 79968-0513, USA.

5 Department of Physics, University of Illinois at Chicago, Chicago, Illinois 60607, USA.

6 Department of Biopharmaceutical Sciences, University of Illinois at Chicago, Chicago, Illinois 60612, USA.

7 Institute of Technical and Macromolecular Chemistry, RWTH Aachen University, Worringerweg 2, 52074 Aachen, Germany.

[#]These authors contributed equally to this work.

*To whom correspondence should be addressed. E-mail: zhenglifei0926@gmail.com; herrmann@dwi.rwth-aachen.de

Table of contents

1. General and materials ……………………………………………………...3

2. Isothermal Titration Calorimetry (ITC) analyses ……………….………4

3. Synthesis and characterization of F-dimers ……………………..…….5

4. Molecular Dynamic Simulations …………………..……………..….12

5. Determination of MIC values of F-dimers ………………….………14

6. Photochemical control over GFP expression with F-dimer …………15

7. Reference ……………………………………………………..…….19

1. General and materials

1D NMR and heteronuclear single-quantum correlation (HSQC) spectra were recorded on a Varian Unity Inova (500 MHz for ^{1}H-NMR and125 MHz for ^{13}C-NMR) and Oxford AS400 (400 MHz for ^{1}H-NMR and 100 MHz for ^{13}C-NMR) NMR spectrometer at 25 °C. High resolution mass spectrometry (HRMS) was carried out on a LTQ ORBITRAP XL instrument (Thermo Scientific) employing electron impact ionization in positive ion mode (EI^{+}). Absorption spectra were measured on a spectrophotometer (Thermo Electron, Evolution 300 BB), which is connected with fiber-coupled LEDs with different wavelengths (THORLABS M455F1, M530F2). Chromatographic separations were performed on a *Shimadzu* VP series HPLC modular system (DGU-14A3 Online Vacuum-Degasser, two LC-20 AT pumps, SIL-20A auto sampler, CTP-20 A column oven, RID-10 refractive detector, FRC-10 A fraction collector and Shimadzu LC solution software). HPLC purification was carried out with a Waters Spherisorb ODS-2 C18 analytical (250 x 4.6 mm) and semi-preparative column (250 x 10mm) (spherical particles of 5 μm and 80 Å pore size) using isocratic elution at 20 °C.

All chemicals and reagents were purchased from commercial suppliers and used without further purification, unless otherwise noted. Paromomycin sulfate salt (98 %) was purchased from Sigma Aldrich and used as received. For reversed phase HPLC (rp-HPLC) purification, trifluoroacetic acid (TFA) (Sigma-Aldrich, HPLC grade) and acetonitrile (Sigma-Aldrich, HPLC grade) were used. Ultrapure water (specific resistance > 18.4 MΩ cm) was obtained by Milli-Q water purification system (Sartorius®). Chromatographic separations were performed using a Shimadzu VP series high performance liquid chromatography (HPLC) modular system (DGU- 14A3 Online Vacuum-Degasser, two LC-20 AT pumps, SIL-20A auto sampler, CTP-20 A column oven, RID-10 refractive detector, FRC-10 A fraction collector and Shimadzu LCsolution software). HPLC purifications were performed with a Gemini NX-C18-semi-preparative column (250 x 10 mm, spherical particles of 5 μm and 110 Å pore size) using isocratic elution at 30 °C. Solvent A: H_2O (HPLC grade) + 0.028% v/v trifluoroacetic acid (TFA); solvent B: H_2O/MeCN 2:1+ 0.028% v/v TFA; detection at 314 nm and 275 nm (if not stated otherwise).

2. Isothermal Titration Calorimetry (ITC) analyses

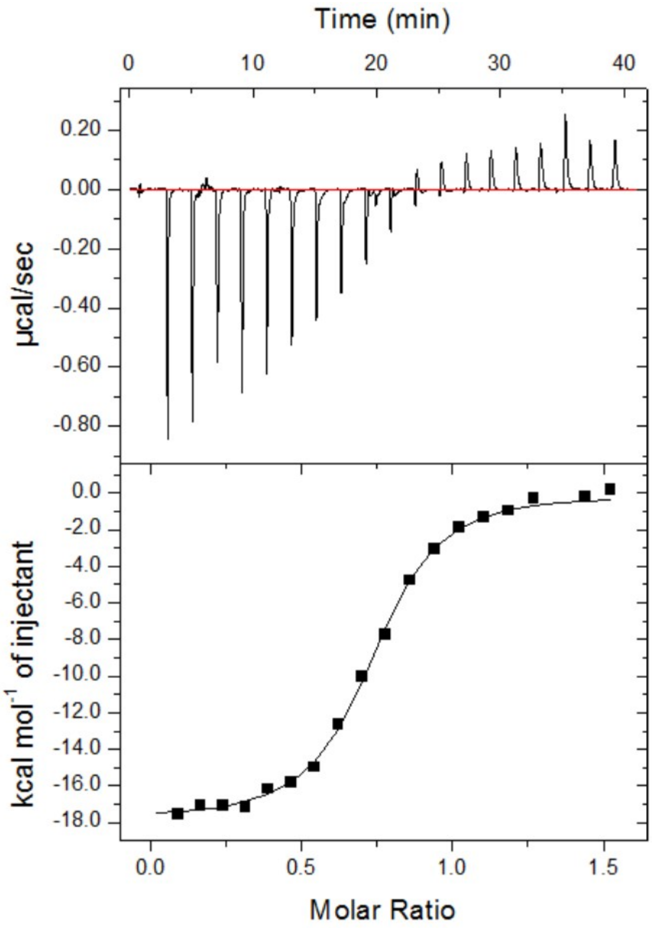

N 0.717 ± 0.0045 Sites

K 4.87E6 ± 1.44E5 M-1

ΔH -1.791E4 ± 158.7 cal/mol

ΔS -31.5 cal/mol/deg

Figure S1. ITC titration curve of paromomycin (70 µM in 20 mM phosphate buffer pH 7.5) into aptamer solution (7 µM in 20 mM phosphate buffer pH 7.5); K_d = 0.2 µM.

3. Synthesis and characterization of F-dimer [1]

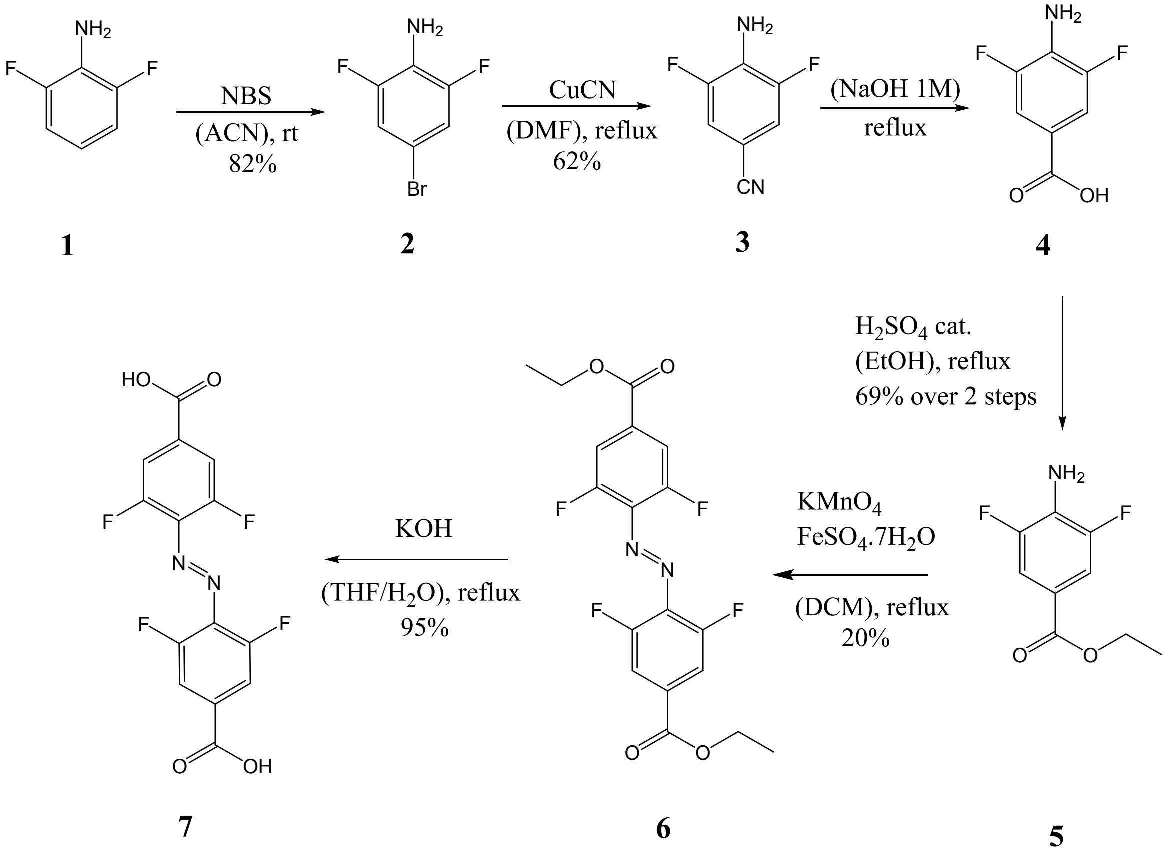

Scheme S1. Synthetic route of F4-diacid (**7**).

2,6-difluoro-4-bromoaniline (2).

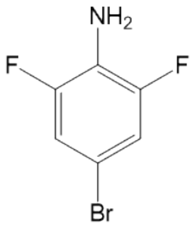

NBS (35.6 g, 200 mmol) was added to a solution of 2,6-difluoroaniline (**1**) (25.8 g, 200 mmol) in acetonitrile (300 mL) and stirred overnight at room temperature. The mixture was diluted with water and hexane. After separation of the layers, the organic phase was dried over $MgSO_4$, filtrated and the solvent was evaporated *in vacuo*. The resulting mixture was purified by column chromatography (DCM/hexanes : 1/1) to give **2** as a purple solid (33.5 g, 80%). ^{1}H NMR (300 MHz, $CDCl_3$) δ ppm 7.00 (dd, J = 6.1, 1.3 Hz, 2 H), 3. 75 (br s, 2 H). ^{13}C NMR (75 MHz, $CDCl_3$) δ ppm 153.36, 150.12, 123.50, 114.72, 107.09.

4-amino-3,5-difluorobenzonitrile (3).

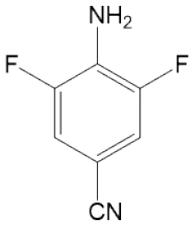

Compound **2** (20 g, 96 mmol) and CuCN (25.6 g, 288 mmol) were dissolved in DMF (100 mL). The mixture was refluxed overnight. After the solution was cooled down, the mixture was poured into a NH_3 12% aqueous solution and extracted with ethyl acetate. The organic phase was dried over $MgSO_4$, filtered, and the solvent was evaporated *in vacuo*. The product was purified by column chromatography (DCM/hexanes : 2/1) to give **3** as white solid (9.2 g, 62%). ^{1}H NMR

(300 MHz, $CDCl_3$) δ ppm 7.16 (dd, *J* = 6.1, 2.2 Hz, 2 H), 4. 29 (br s, 2 H).

4-amino-3,5-difluorobenzoic acid (4).

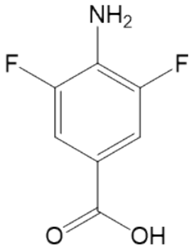

Compound **3** (6.9 g, 45 mmol) was suspended in NaOH 1M (150 mL) and refluxed overnight. After the solution was cooled down, 1M HCl was added until hydrochloric salt **4** precipitated. The precipitate was then dissolved in EtOAc, dried over $MgSO_4$, filtered, and the solvent was evaporated *in vacuo*. The product was obtained as white solid and used without purification in the next step. ^1H NMR (300 MHz, DMSO-*d6*) δ ppm 7.40 (dd, *J* = 4.2, 1.4 Hz, 2 H), 3.81 (br s). ^{13}C NMR (75 MHz, DMSO-*d6*) δ ppm 165.96, 150.65, 148.74, 130.65, 115.59, 112.22. HRMS-ESI: *m/z* = 172.0278 (calcd for $[M - H]^-$, 172.0204).

Ethyl 4-amino-3,5-difluorobenzoate (5).

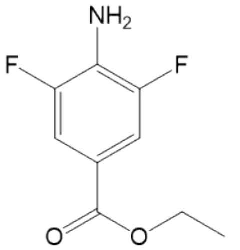

To a solution of acid **4** (5.92 g, 28 mmol) in EtOH (100 mL) was added H_2SO_4 (2 mL) and refluxed for 5 h. Saturated $NaHCO_3$ was added until the solution was neutralized (pH 7). The mixture was extracted with DCM and the organic phase was dried over $MgSO_4$ and filtered. The solvent was evaporated under reduced pressure to give **5** as a pale brown solid (4.2 g, 69% over two steps). ^1H NMR (300 MHz, $CDCl_3$) δ ppm 7.50 (dd, *J* = 7.2, 2.2 Hz, 2 H), 4.30 (q, *J* = 7.1 Hz, 2 H), 4.07 (br s, 2 H), 1.34 (t, *J* = 7.1 Hz, 3H). ^{13}C NMR (75 MHz, $CDCl_3$) δ ppm 165.19, 152.21, 149.03, 128.78, 118.38, 112.55, 61.04, 14.25. HRMS-ESI: *m/z* = 202.0535 (calcd for $[M + H]^+$, 202.0680).

Diethyl-4,4'-(2,2',6,6'-tetrafluoro)azobenzene dicarboxylate (6).

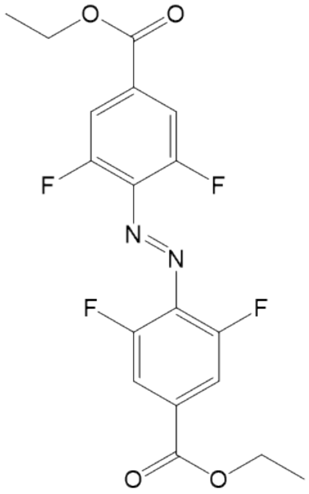

Compound **5** (600 mg, 3 mmol) was dissolved in DCM (100 mL) and a freshly grinded mixture of $KMnO_4$ (5.1 g) and $FeSO_4 \cdot 7H_2O$ (5.1 g) was suspended in this solution. The mixture was refluxed overnight, filtered through celite, dried over $MgSO_4$, filtered, and the solvent was evaporated *in vacuo*. The product was purified by column chromatography (DCM/hexanes : 1/1) to give **6** as an orange/red solid (136 mg, 23%). ^1H NMR (300 MHz, $CDCl_3$) (*E*-isomer) δ ppm 7.76 (m, 4 H), 4.45 (q, *J* = 7.0 Hz, 4 H), 1.45 (t, *J* = 7.0 Hz, 6 H). ^{13}C NMR (75 MHz, $CDCl_3$)(*E*-isomer) δ ppm 163.63, 156.75, 153.24, 133.80, 113.90, 62.18, 14.20. HRMS-ESI: *m/z* = 399.0999(calcd for $[M + H]^+$, 399.0968).

F4-diacid (7).

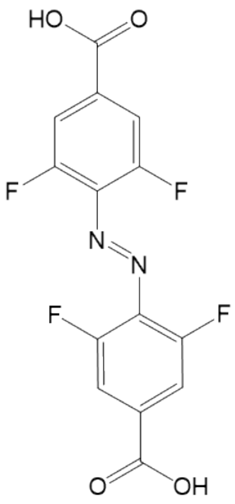

Compound **F4-diester 6** (136 mg, 0.34 mmol) and KOH (67 mg, 1.19 mmol) were dissolved in H_2O/THF 2:1 (4 mL) and the solution was heated at 70 °C for 2 h. The solution was diluted with water and ethyl acetate, the two layers were separated, and the aqueous layer was washed twice with ethyl acetate. The aqueous layer was acidified with HCl (1M) until the orange precipitate fell out from solution. The orange precipitate was filtered to yield **7** as an orange solid (113 mg, 97%). HRMS-ESI: *m/z* = 342.0264. (calcd for $[M - H]^-$, 342.2056).

Into a solution of paramomycin sulfate salt (0.3 mmol) in water/methanol (1:1 10 mL), ion-exchange resin was added and the solution was stirred for 2 h at room temperature. Ion-exchange resin was removed by filtration and the aqueous solution was concentrated under reduced pressure to remove methanol and freeze dried.

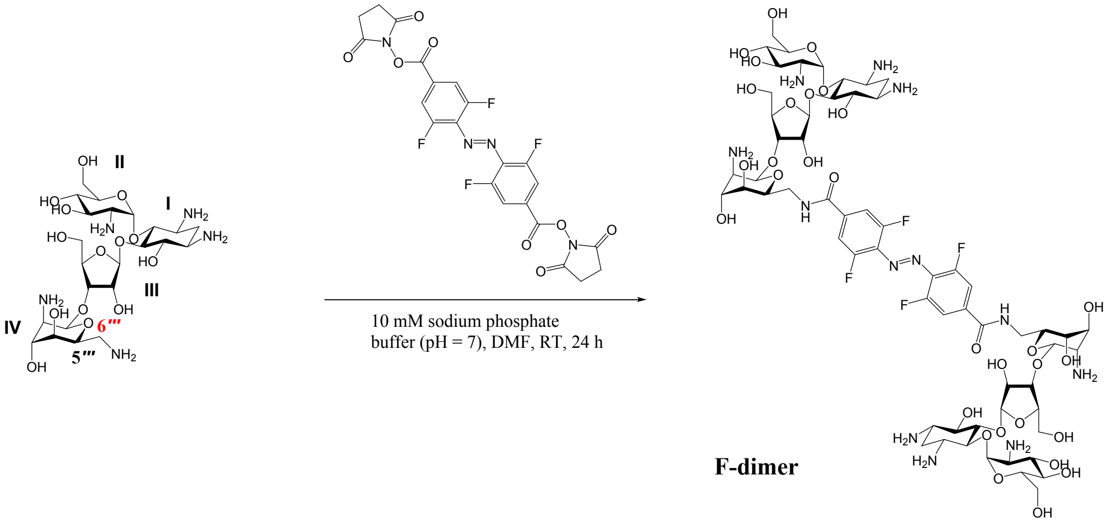

Scheme S2. Synthetic route of **F-dimer**.

F-dimer

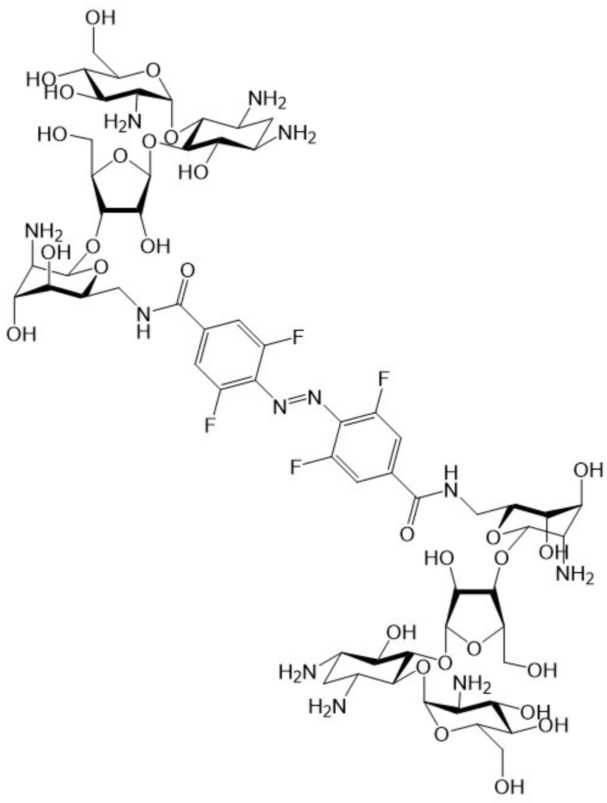

In a 5 ml round bottom flask, F4-diacid **7** (20.4 mg, 0.06 mmol) and N- hydroxysuccinimide (13.8 mg, 0.12 mmol) were dissolved in 20 mL dry DMF. After the addition of N,N'-dicyclohexylcarbodiimide (24.8 mg, 0.12 mmol), the reaction was continued for 12 h under inert atmosphere at room temperature. Precipitated dicyclohexylurea (DCU) was removed by filtration. F4-diacid-NHS was used for coupling without further purification.

Paromomycin (0.3 mmol) was dissolved in 20 mM sodium phosphate buffer (20 mL, pH 7.0), a solution of **F4-diacid**-NHS in DMF (2.5 mL) was added drop by drop in 3 h. The reaction was carried out overnight under inert atmosphere at room temperature. The solvent was evaporated. The obtained crude mixture was purified by column chromatography by using homogeneous mobile phase consisting of dichloromethane/methanol/aq. 25% ammonia (from 2:2:1 to 2:3:2 v/v/v) mixture. After evaporation of the solvent, the residue was re-dissolved in water (3 mL) and traces of silica were removed by filtration through a 0.45 mm-syringe filter. Lyophilization yielded the **F-dimer** as orange solid. Yield: 51.2 mg (0.03 mmol, 56%). ^{1}H NMR (500 MHz, D_2O) (*E*-isomer) δ ppm 7.69 (m, 4 H, azo), 5.73 (d, J = 3.5 Hz, 2H, 1-H´), 5.40 (d, J = 1.5 Hz, 2H, 1-H´´), 5.27 (s, 2H, 1-H´´´), 4.44 (t, J = 5.5 Hz, 2H, 3-H´´), 4.34 (m, 2H, 2-H´´), 4.31 (m, 2H, 3-H´´´), 4.26 (t, J = 5.5 Hz 2H, 5-H´´´), 4.22 (m, 2H, 4-H´´), 4.01 (t, J= 7.8 Hz, 2H, 4-H), 3.92-3.82 (m, 10H, 6a-H´´´, 5a-H´´, 6a-H´, 3-H´, 5-H), 3.81-3.68 (m, 8H, 5b-H´´, 5-H´, 6b-H´, 4-H´´´), 3.72 (t, J = 9.5 Hz, 2H, 6-H), 3.64 (m, 2H, 6b-H´´´), 3.60(m, 2H, 2-H´´´), 3.58 (t, J = 9.75 Hz, 2H, 3-H), 3.44 (t, J = 7.75 Hz, 2H, 4-H´), 3.40 (dd, J = 11.0 Hz, J = 4.0 Hz, m, 2H, 2- H´), 3.37(m, 2H, 1-H), 2.50 (dt, J = 13.0 Hz; J = 4.0 Hz, 2H, 2-He), 1.86 (dd, J = 12.0 Hz, 2H, 2-Ha). ^{13}C-signals based on HSQC (D_2O, 500 MHz) δ ppm 112.23 (azo), 109.65 (C-1´´), 92.13 (C-1´), 96.024 (C-1´´´), 84.12 (C-5), 81.98 (C- 4´´), 77.60 (C-4), 76.32 (C-3´´), 73.91 (C-5´), 73.87 (C-2´´), 72.48 (C-5´´´), 72.23 (C-6), 69.26 (C-4´), 68.82 (C-3´), 67.68(C-3´´´), 66.34 (C-4´´´), 60.37 (2C, C-5´´, C-6´), 53.76 (C-2´), 51.09 (C-2´´´), 49.61 (C-1), 48.82 (C-3), 40.29 (C-6´´´), 28.08 (C-2). HRMS-ESI: m/z = 1537.62; 769.48 (calcd for $[M+H]^{+}$, 1537.60; $[M+2H]^{2+}/2$=769.30).

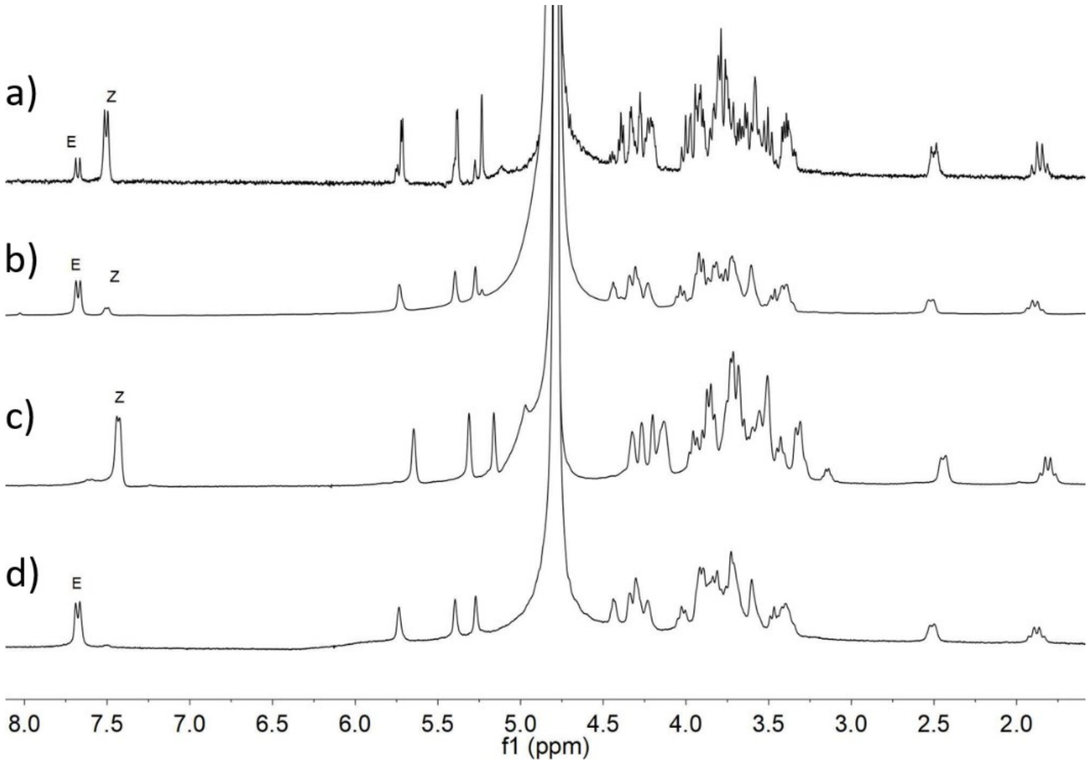

Figure S2. ^{1}H-NMR (500MHz, D_2O) spectrum of F-dimer. a) PSS after green light (>500 nm) irradiation; b) PSS after blue light (455 nm) irradiation; c) pure *Z*-F-dimer after HPLC purification; d) pure *E*-F-dimer after HPLC purification. (The peak at 4.79 ppm is the solvent peak of D_2O.)

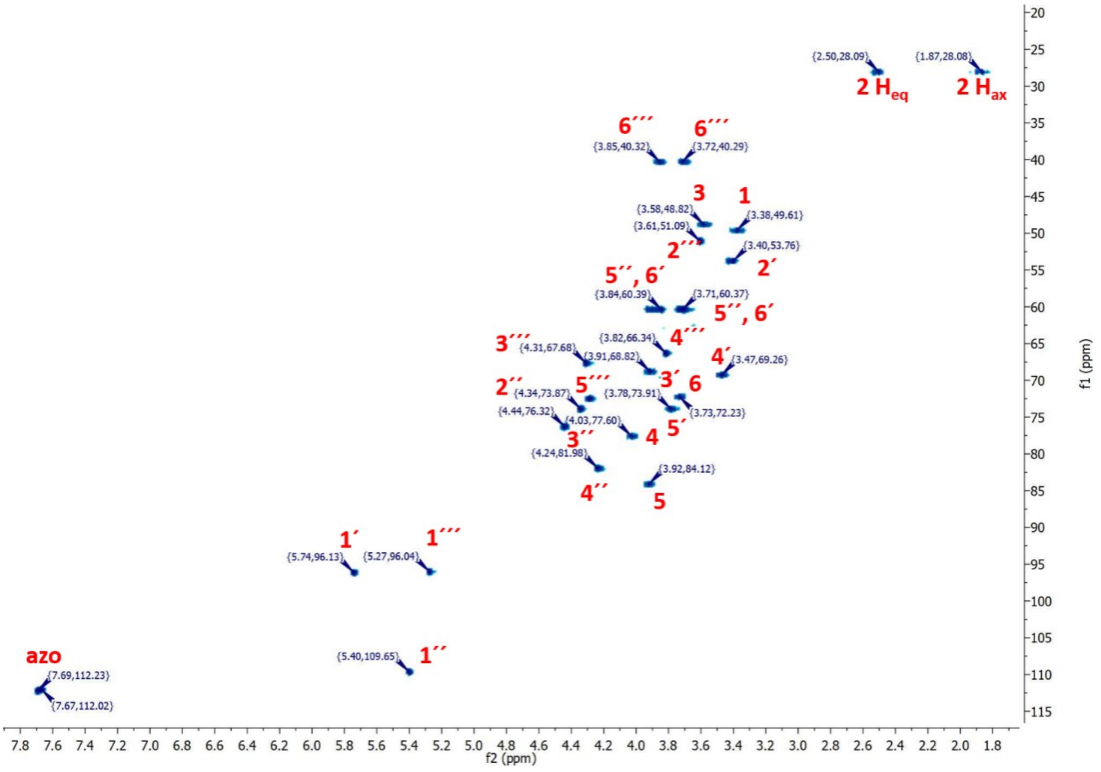

Figure S3. HSQC spectrum (500 MHz, D_2O) of F-dimer.

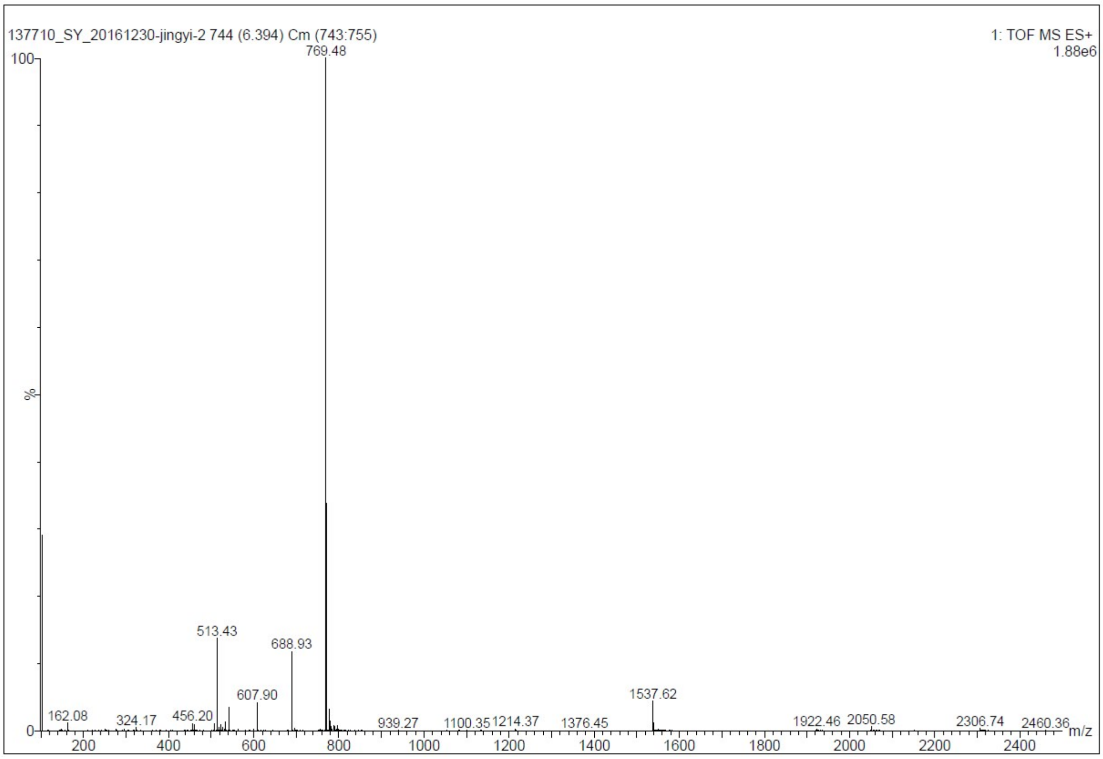

Figure S4. HRMS-ESI analysis of F-dimer.

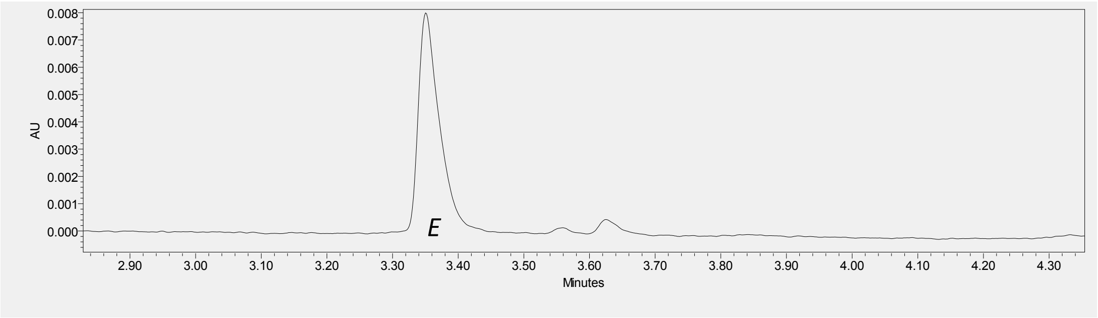

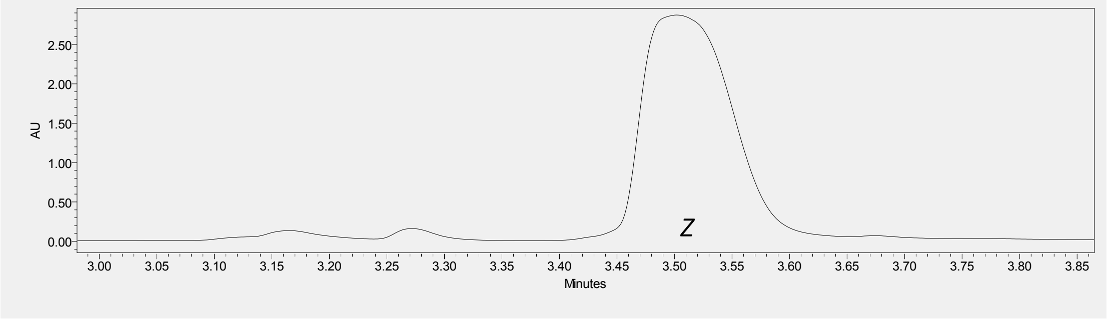

Figure S5: UPLC traces (recorded at the corresponding isosbestic points) of purified *E*- and *Z*-isomer.

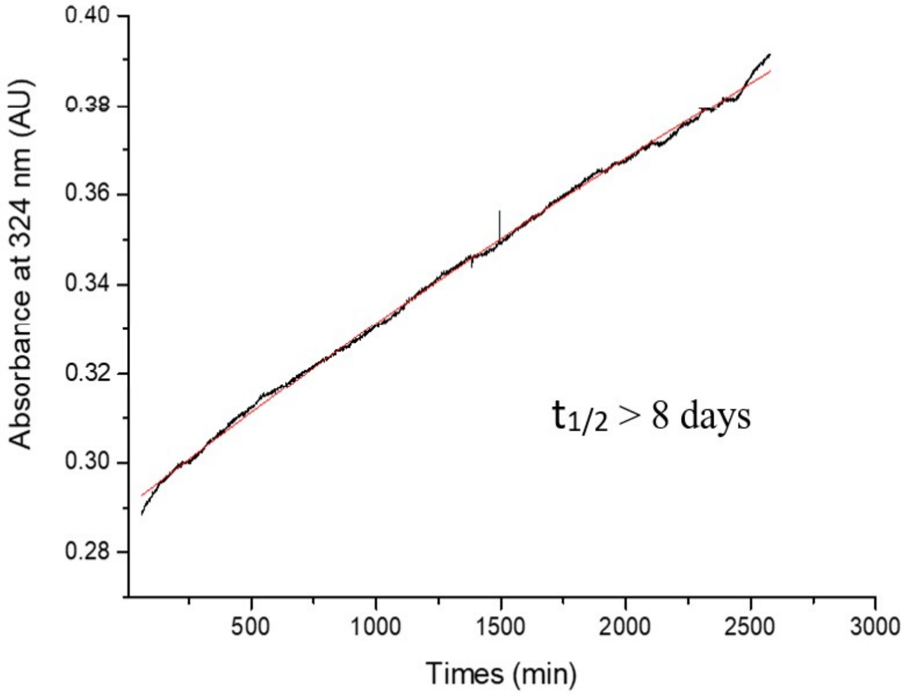

Figure S6. Determination of thermal half-life for F-dimer at 37 °C in water in the dark. First, PSS was reached upon >500 nm irradiation, after which the absorption was measured at λ_{max} = 324 nm.

4. Molecular Dynamic Simulations

The structure of RNA aptamer (23 nucleotides) was taken from the PDB and is based on a published NMR structure. [2] The *E*/*Z*-isomers and RNA aptamer were simulated by NAMD [3] and CHARMM [4, 5] force fields. The particle mesh Ewald (PME) [6] method was used for the evaluation of long-range Coulombic interactions. The time step was set to 2 fs. The simulations were performed in the NpT ensemble (p = 1 bar and T = 310 K), using Langevin dynamics with a damping constant of 1 ps^{-1}. After 2000 steps of minimization, ions and water molecules were equilibrated for 2 ns around RNA and E/Z-isomers, which were restrained using harmonic forces with a spring constant of 2 kcal/(mol $Å^2$). The last frames of restrained equilibration were used to start simulations of free molecules and RNA aptamer. The simulations last for 1.9–2 µs. The calculations of binding energy and RMSD were performed by VMD plugin. [7] The binding energy is composed of vdW and electrostatic energy, where the dielectric constant is set to 1 during the calculation.

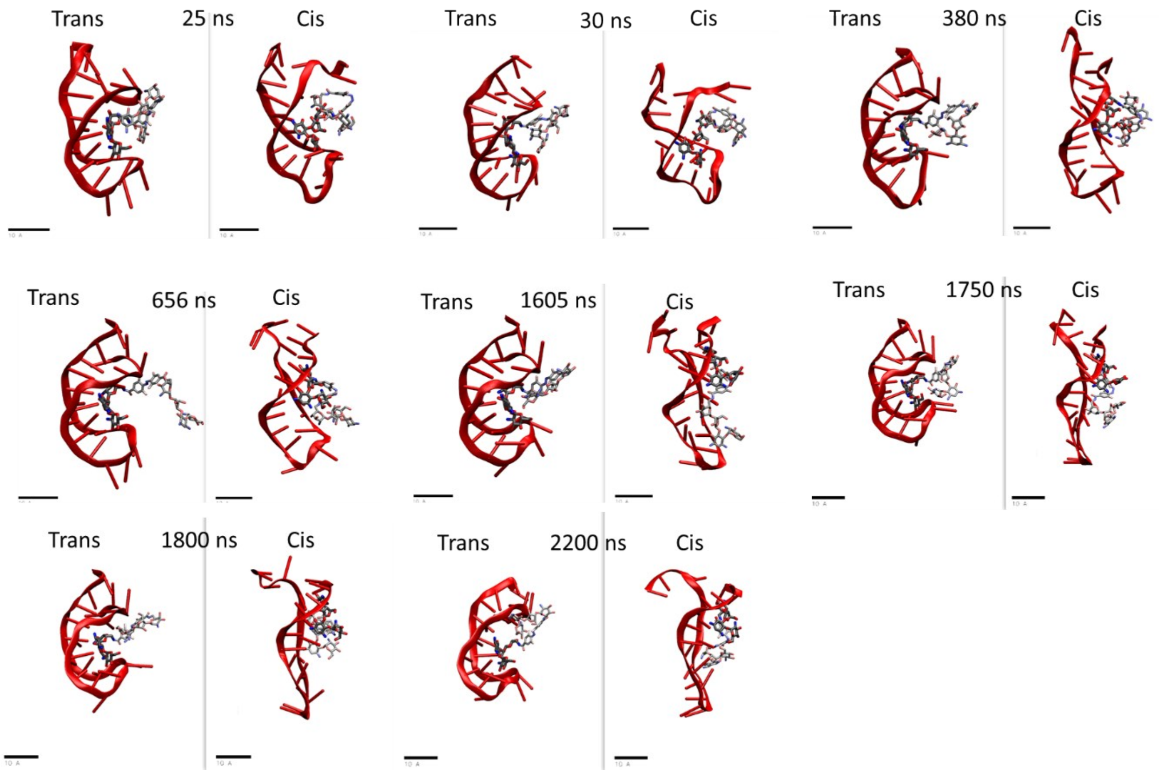

Figure S7. Trajectories of F-dimers interacting with RNA aptamer.

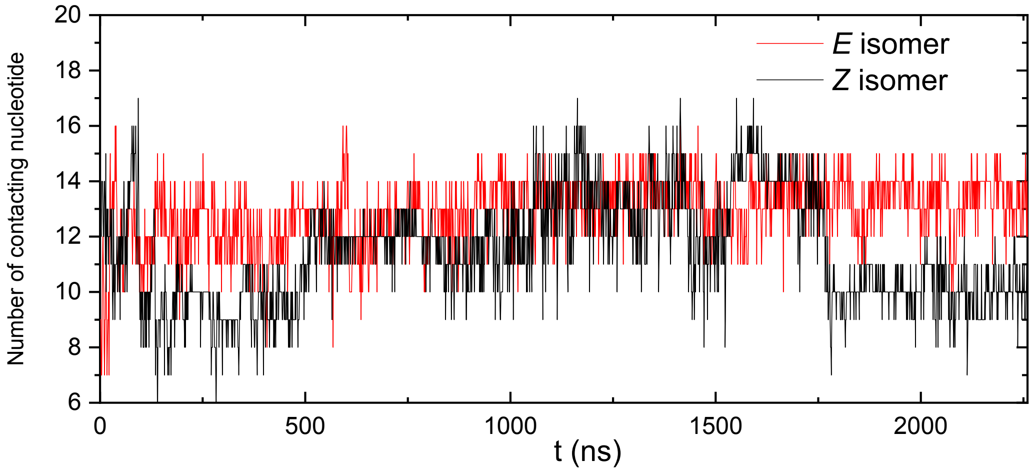

Figure S8. Number of contacting nucleotides with F-dimers.

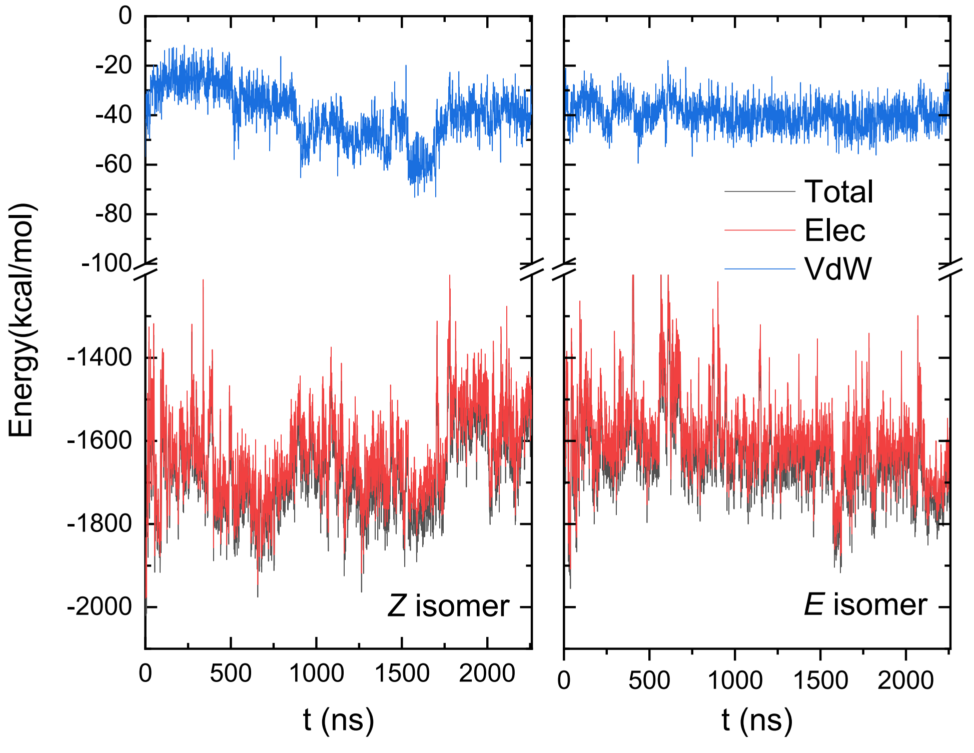

Figure S9. Binding energy between Z/E-isomers and RNA aptamer.

5. Determination of MIC values of F-dimers

The F-dimers were serial diluted from 1024 μm to 16 μm in 15 mL tubes. Overnight cultures of E. coli ATCC 25922 were diluted to an optical density at 600 nm of 0.1 and 100 μl of this cell suspension was added to total volume of 500 μl LB medium containing the F-dimers at the given concentration. After overnight growth at 37°C and shaking at 220 rpm, the optical density at 600 nm was measured. Graphs were background-corrected by subtracting the OD of the LB medium without any bacteria.

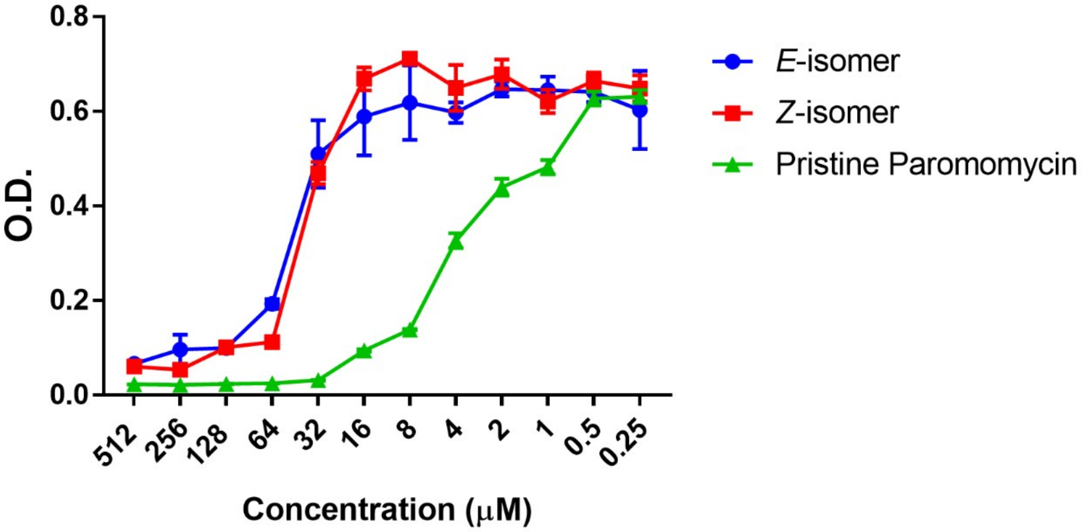

Figure S10. MIC test of the *E* and *Z* F-dimers to determine the working concentration for gene switching experiments. Pristine paromomycin was added as reference.

6. Photochemical control over GFP expression with F-dimers

Growth of the E. coli cells and expression of the TMS switch as well as the proteins. The conditions of growth of the E. coli BL21(DE3) cells and the expression of the proteins along with the switch was maintained in the same way as it was described in previous work 8. To grow the bacteria on the plates, culture from the logarithmic growth phase was spread on the agar plates. The plates contain appropriate antibiotics (ampicillin 50 µg/ml and chloramphenicol 25 µg/ml) and the inducers to express the switch and GFP (1 mM IPTG to express the switch and 0.1 % arabinose (w/v) to express the proteins). After spreading the cells, depending on the experimental conditions, the plates were either kept overnight at 37°C or were exposed to light and then kept overnight at 37°C.

Flow cytometry measurements. Flow cytometry measurements were performed on a BD FACS Canto flow cytometer. The BD FACS Canto was calibrated with CST beads from BD Biosciences (Cat No: 655051). The bacterial plates were treated with 1X PBS to disperse the bacterial colonies into the PBS buffer and were diluted by 100-fold into fresh 1X PBS buffer prior measurement. For GFP fluorescence measurement, a 488 nm excitation filter (optical power 20mW) and 515-545 nm emission filter were used. For each sample, 50.000 events were recorded. All the samples were measured with low sample flow rate (approximately 12 µl/min). The flow cytometry analysis was performed using FlowJo software (version 10). Note: ON/OFF ratios were calculated based on the positive population only.

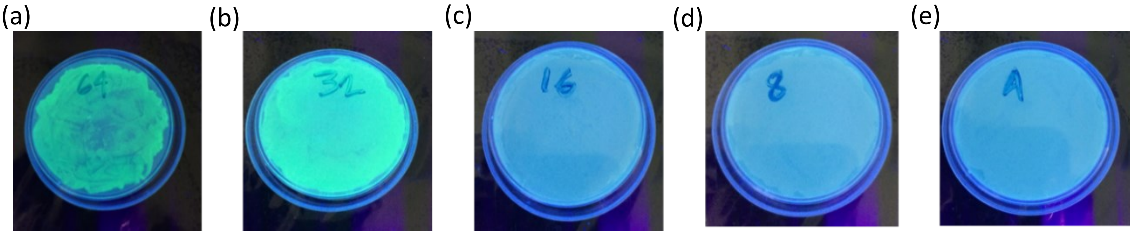

Figure S11. The effect of the concentration of the *E*-isomer on the GFP expression. (a) GFP expression with 64 µM E-isomer; (b) GFP expression with 32 µM E-isomer; (c) GFP expression with 16 µM E-isomer; (d) GFP expression with 8 µM E-isomer; (e) GFP expression with 4 µM E-isomer.

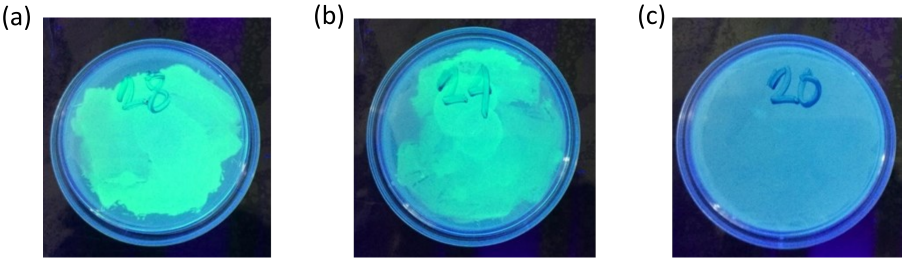

Figure S12. The effect of the concentration of the *E*-isomer on the GFP expression. (a) GFP expression with 28 µM E-isomer; (b) GFP expression with 24 µM E-isomer; (c) GFP expression with 20 µM E-isomer.

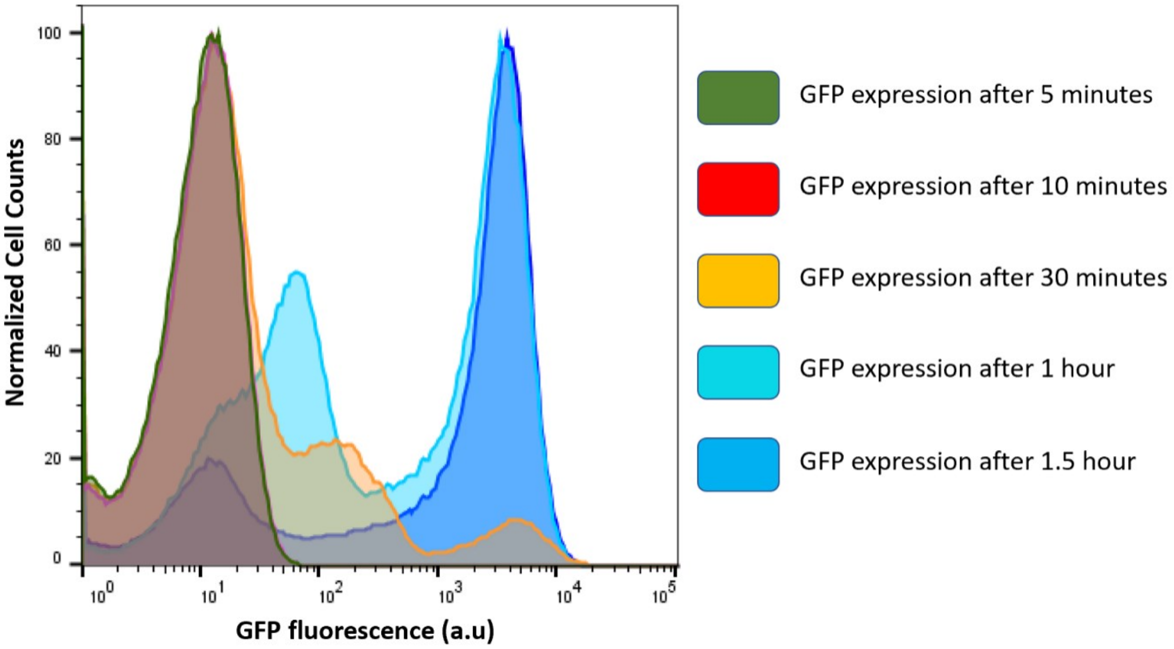

Figure S13. *In situ Z* to *E* photo-isomerization induced switch-on of GFP expression under the irradiation at 455 nm for different times.

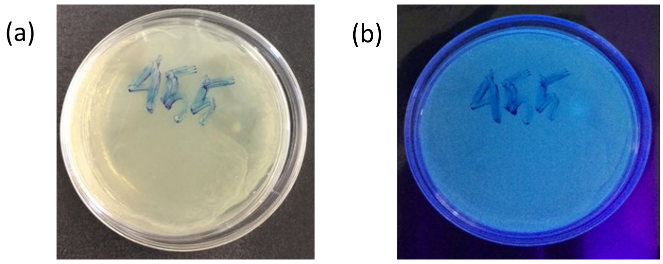

Figure S14. The effect of light (455 nm) on GFP expression. (a) Bright field image showing complete bacterial growth on the plate; (b) Image of the plates under UV lamp. It was observed that no GFP expression occurred with the treatment of light only.

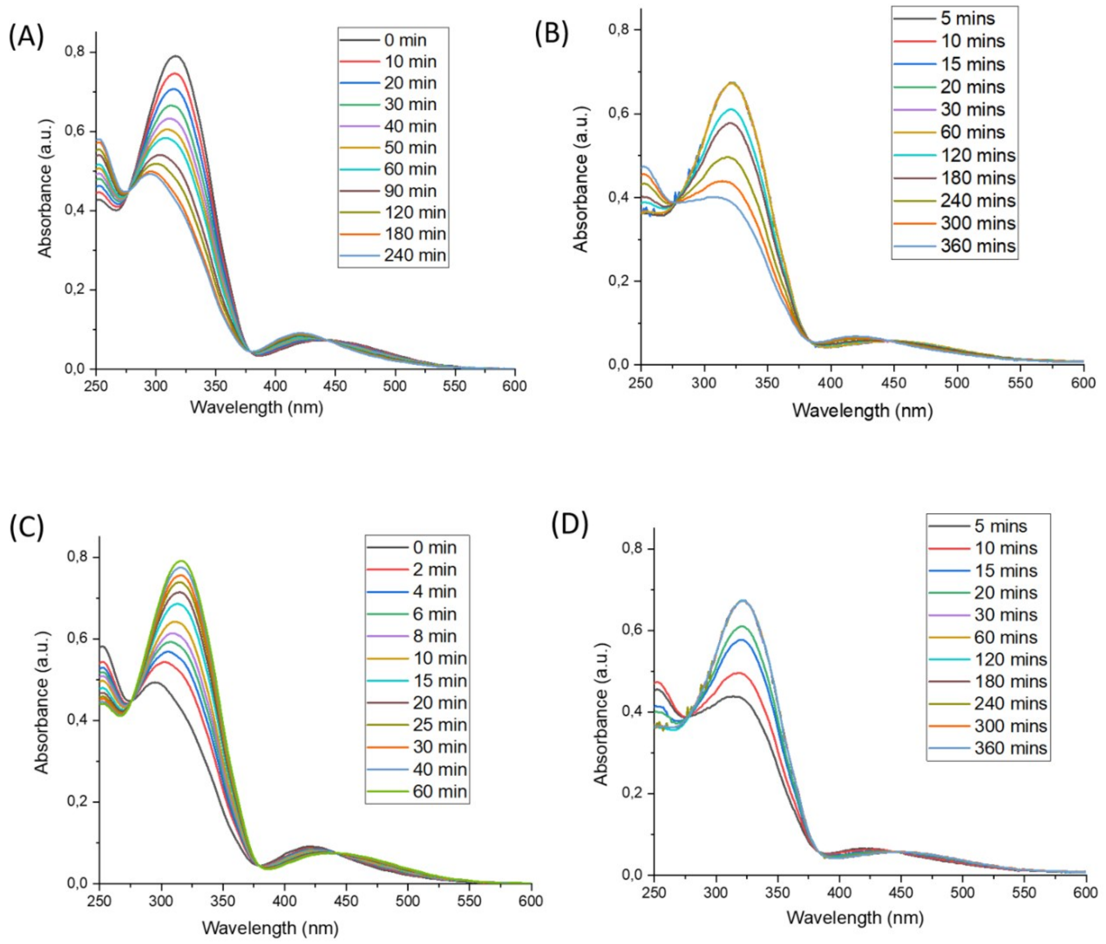

Figure S15. Changes in the absorption spectra of a solution of F-dimer (10 μM): upon irradiation at 530 nm (*E*→*Z*) in the absence (A) and in the presence of RNA aptamer (B); upon irradiation at 455 nm (*Z*→*E*) in the absence (C) and in the presence of RNA aptamer (D).

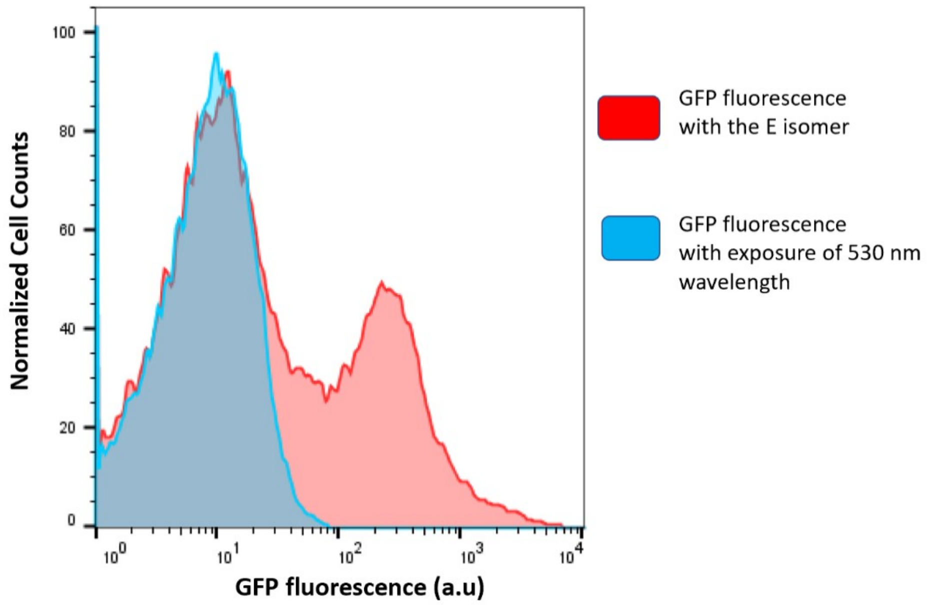

Figure S16. *In situ E* to *Z* photo-isomerization induced switch-off of GFP expression under the irradiation at 530 nm for 1.5h.

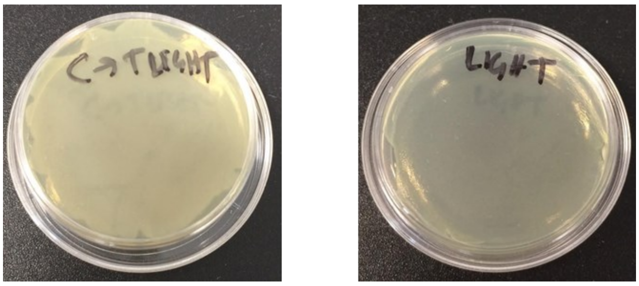

Figure S17. (a) Bright field image of the plate containing *Z* isomer only but without adding the inducer to express the lysis gene; (b) Bright field image of the plate containing the inducers to express the TMS switch and the lysis gene but without adding *Z* isomer.

7. Reference

[1] C. Knie, M. Utecht, F. Zhao, H. Kulla, S. Kovalenko, A. M. Brouwer and P. Saalfrank, *Chem. Eur. J.*, 2014, **20**, 16492–16501.

[2] L. Jiang, A. Majumdar, W. Hu, T.J. Jaishree, W. Xu and D.J. Patel, *Structure,* 1999, **7**, 817-827.

[3] J. C. Phillips, R. Braun, W. Wang, J. Gumbart, E. Tajkhorshid, E. Vilia, C. Chipot, R.D. Skeel, L. Kale and K. Schulten, *J. Comput. Chem.,* 2005, **26**, 1781-1802.

[4] K. Hart, N. Foloppe, C.M. Baker, E.J. Denning, L. Nilsson and A.D. MacKerell, *J. Chem. Theory Comput.,* 2012, **8**, 348-362.

[5] K. Vanommeslaeghe, E.P. Raman and A.D. MacKerell, *J. Chem. Inf. Model.,* 2012, **52**, 3155-3168.

[6] T. Darden, D. York and L. Pedersen, *J. Chem. Phys.,* 1993, **98**, 10089-10092.

[7] W. Humphrey, A. Dalke and K. Schulten, *J. Molec. Graphics,* 1996, **14**, 33-38.

[8] A. Paul, E.M. Warszawik, M. Loznik, A.J. Boersma and A. Herrmann, *Angew. Chem. Int. Ed.,* 2020, doi.org/10.1002/anie.202001372.
